# Subjective Importance of a Common Feature Decides Its Consideration in Multi-attribute Decision-making

**DOI:** 10.3389/fpsyg.2020.583999

**Published:** 2021-01-14

**Authors:** Ziyi Wang, Guibing He

**Affiliations:** Department of Psychology and Behavioral Sciences, Zhejiang University, Hangzhou, China

**Keywords:** multi-attribute decision-making, common feature, attention, top-down, environmental decision-making

## Abstract

One of the interesting research questions in multi-attribute decision-making is what affects the consideration of shared information (i.e., common features) between two alternatives. Previous studies have suggested two approaches (bottom-up and top-down) in finding what characteristics of common features affect their consideration. Two bottom-up factors (salience and interdependence) were found, but no top-down factors were discovered. In the current study, we followed the top-down approach and investigated how subjective importance (SI) of a common feature affects its consideration. In two studies, we consistently found that, on both the general and individual level, the level of consideration increased with the SI of the common feature. This result provided a new explanation for the effect of common feature consideration and its individual difference; it also provided insights in explaining the underlying process of multi-attribute decision making.

## Introduction

People have a natural tendency to focus on things that are unique and ignore those that are common (Kahneman and Thaler, 2006). When choosing between two multifeature alternatives, it is often convenient and effort-saving for people to cancel their common features (i.e., features sharing the same content among alternatives) and pay more attention on the unique ones (i.e., features having different contents, Kahneman and Tversky, 1979; Houston and Sherman, 1995; Houston and Roskos-Ewoldsen, 1998).

However, cancelation may not always happen. The content from common features may still be considered and has impact on the decision. For example, when choosing between two bottles of milk with different prices and volumes (like a $2, 1-liter bottle and a $5, 1-gallon bottle), their shared expiration date can alter the decision. When the expiration date is far, people may choose the cheaper, 1-gallon bottle, but when it is near, people may favor the more expensive, 1-liter bottle to avoid potential waste. This indicates that some common features still matter, but what characteristics of common features make them considered in a decision? In this research, we investigated the question and suggested that, the level of consideration can be decided by subjective importance (SI) of the common features in the decision.

The whole paper consists of four major sections. In the first section, we reviewed previous studies focusing on the effect of common feature consideration and raised our research question. In the second section, we provided Study 1 which investigated the effect of SI of common features on both the general and the individual level. In the third section, we provided Study 2 which replicated our result in Study 1 and further investigated this effect more directly. In the fourth section, we discussed our findings and provided the limitations of the current study as well as future research orientations.

### Are Common Features Canceled?

Studies in the literature have produced mixed results on whether common features are canceled in the process of decision-making (Tversky, 1972; Houston and Sherman, 1995; Chernev, 2001; Li et al., 2007; Su et al., 2012). Some researchers proposed that common features should have no effect on judgment of alternatives and therefore are canceled to simplify mental representation of the choice (Tversky, 1972; Kahneman and Tversky, 1979). Accordingly, a model called cancelation-*and-focus* was proposed to describe the process (Houston et al., 1989, 1991; Houston and Sherman, 1995). This model asserts that common features are first canceled and greater weight is placed on unique features of alternatives (Houston and Sherman, 1995; Houston and Roskos-Ewoldsen, 1998). Experimental studies later provided evidence to support this model (Dhar and Sherman, 1996; Hodges, 1997, 1998). For example, Hodges (1997) found that participants rated both apartments with shared positive and unique negative features higher when the options were presented separately than together, which means the same positive feature received less attention (i.e., canceled), when serving as a common feature in the joint presentation.

Other researchers proposed that common features with certain qualities should be considered (i.e., not canceled) and can still affect the result of a decision (Chernev, 2001; Li et al., 2007; Su et al., 2012; Du and MaCdonald, 2015). For example, Li et al. (2007) found that adding a common feature to both alternatives could reverse participants’ original preferences. Moreover, it was found that, compared to irrelevant common features, only relevant ones (i.e., when common features fall in the same dimension of a unique feature) could elicit the reversal. This result was interpreted by a model called *equate-to-differentiate* (Li, 2001, 2003), which asserts that, when a common feature is relevant to unique features, its addition can increase or decrease subjective difference of the unique features between two alternatives, hence changing individuals’ preferences. For instance, when choosing a fast food combo, the addition of an extra beverage can lessen the subjective difference between a large Coke and a small Coke, while the addition of a pack of irrelevant napkin will not do (Li et al., 2007).

### What Characteristics of Common Features Affect the Consideration?

Two major determinants affecting the level of consideration can be concluded from previous studies. One is the *interdependence* between common features and unique features. When valuation of the same unique features varies with the content of common features, the common features and unique features become interdependent, which makes common features considered in decision-making (Li et al., 2007; Su et al., 2012). Interdependence may appear when common features are *relevant* to unique features (e.g., buying fast food combo, common feature: chicken wings; unique feature: hamburger), or when common features have *multiplicative* relation with unique features (e.g., buying lottery, common feature: winning rate; unique feature: prize money).

The other determinant is the *salience* of common features. Common features become salient when they are presented in a distinctive way, which will attract more attention in the process of decision-making and have larger impact on the decision. For example, Slaughter and Highhouse (2003) found that when a common feature was presented in a more salient complex format, rather than a simple format, it affected results of the decisions and had higher level of consideration. Du and MaCdonald (2015) found that the same common features had higher level of consideration when presented with images (i.e., the more salient form) than with texts.

These findings have provided some answers to what characteristics of common features affect their consideration. However, the studied effects were mostly induced by independent characteristics (e.g., relevance and presentation) of the features, which only covered the bottom-up approach in explaining the effect, and left out a crucial factor (i.e., decision makers’ subjective view about common features). Only Chernev (2001) followed the top-down approach and found that common features had higher level of consideration when they are more attractive to the decision maker.

Moreover, previous findings have suggested that individuals’ visual attention should be affected by both the salience of the stimuli (i.e., the bottom-up approach), as well as its SI to the individual (i.e., the top-down approach, Connor et al., 2004; Theeuwes, 2010; Anderson et al., 2011). Therefore, following the top-down approach, we hypothesized that the level of consideration should increase with the SI of the common features. This hypothesis was tested at multiple levels in two studies.

## Study 1

In study 1, we asked participants to rate their relative preference between two multi-attribute environmental protection projects (EPPs), where one of the attributes was set as the common feature. We investigated the effect of SI on the level of consideration by comparing the attention that a common feature received and its impact on the decision, when setting attributes with different SI as the common feature in the two EPPs. The importance of involved attributes (i.e., Monetary Cost [MC], Success Rate [SR], Time Cost [TC], and Environmental Outcome [EO]) have been investigated by several studies (Ramanathan, 2001; Huang et al., 2011; Kiker et al., 2015), which provided references for our manipulation of the SI and for the investigation of its effect.

To cover different aspects of the effect, we tested our hypothesis at two different levels. On the general level, an attribute has higher general SI when most people view it as more important than other attributes. Thus, we tested whether setting an attribute with higher general SI as the common feature would lead to a higher level of consideration. We measured the general SI of all involved attributes in a preliminary study (see Supplementary Materials), and investigated its effect by setting attributes with different level (e.g., low, medium and high) of general SI as the common feature. We assumed that the level of consideration would increase with the level of general SI.

On the individual level, because different people have different favoritism over the attributes, some individual’s SI of an attribute may be inconsistent with its general SI. Therefore, we measured the participants’ individual SI of the common feature, and tested whether a common feature with low general SI would still matter when someone viewed it as relatively more important. We assumed that when controlling the general SI of a common feature, the level of consideration would increase with the individual SI of that common feature.

### Methods

#### Preliminary Study: Measuring General SI of Attributes

**FIGURE 1:**
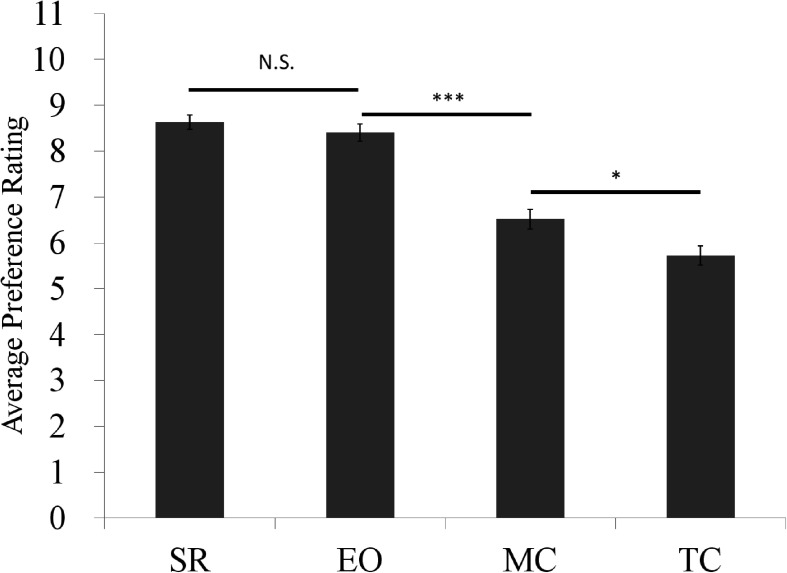
Average importance ratings for attributes (error bar = standard error). N.S. *p* > 0.05, ^∗^*p* < 0.05, ^∗∗∗^*p* < 0.001.

**FIGURE A1:**
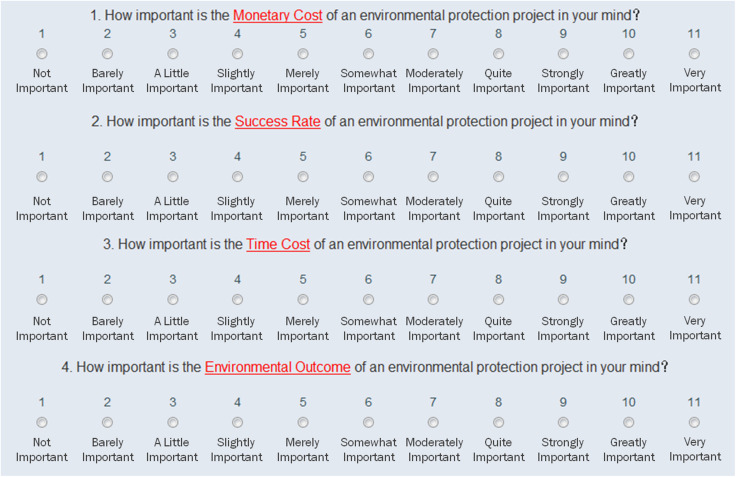
Eleven-point scales used in preliminary study.

A preliminary study was first conducted to predetermine the general SI of different attributes using an independent sample of participants (*N* = 116, female: 51, male: 65, mean age = 23.71, SD = 1.26, see Supplementary Materials). All participants finished a questionnaire online which was posted on the Bulletin Board System (BBS) of Zhejiang University. In the questionnaire, they were asked to rate the importance of MC, SR, TC, and EO on eleven-point scales (see Appendix Figure A1), ranging from “1: Not important” to “11: Very Important.” They were told that the larger the number they choose, the more important they thought that attribute was. Results from pairwise comparisons showed that SR (Mean = 8.64, SD = 1.68) and EO (Mean = 8.41, SD = 1.93) were the most important attributes, MC (Mean = 6.52, SD = 2.26) was the second and TC (Mean = 5.72, SD = 2.24) was the third (see Figure 1).

#### Study 1: Participants and Design

Seventy-three undergraduate students (female: 58, male: 15, mean age = 21.00, SD = 0.85) from a local university (Communication University of Zhejiang) were recruited for this experiment. In exchange for credit, participation was required for all the students in a course teaching the methods of social research. Because this university generally has much more female students (75%) than male students (25%), the female–male proportion in this sample is uneven. This was corrected in Study 2 when recruiting participants in another university.

All participants were asked to finish the experiment on a webpage with their personal computers. A within-subject design was used in this study (Independent variable: General SI of common features [Low SI feature: TC; Medium SI feature: MC; High SI feature: SR], dependent variable: level of consideration of common features).

Based on a power analysis using G^∗^Power (Faul et al., 2007), we determined that to achieve a statistical power of 0.80 with a significance level of 0.05, a medium effect size (*f*^2^) of 0.15, we needed a sample size of 55 participants. Since some data may be invalid, we recruited slightly more participants.

#### Procedure and Material

**FIGURE 2:**
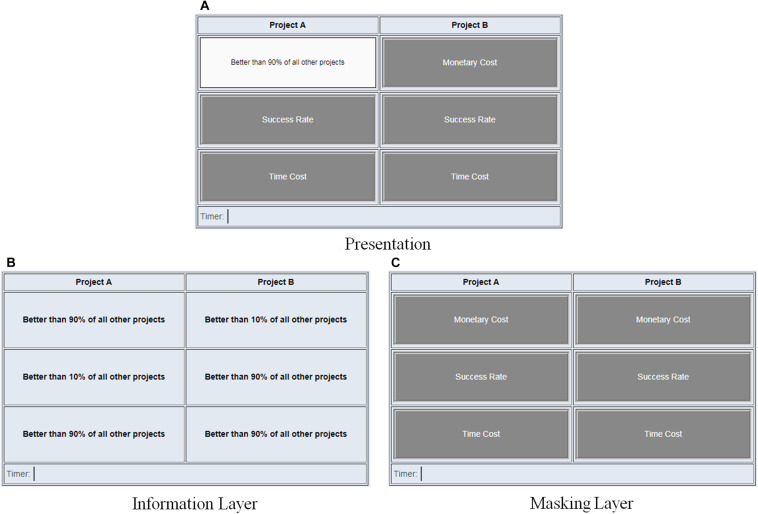
Demonstration of the decision task in Study 1. **(A)** Presentation, **(B)** Information Layer, **(C)** Masking Layer.

**FIGURE A2:**
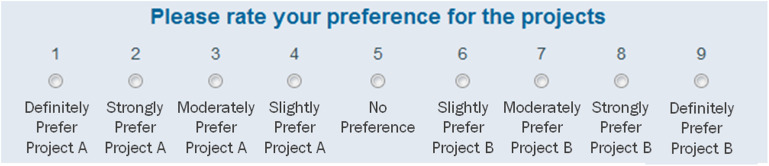
Nine-point scales used in Studies 1 and 2.

In the experiment, participants were asked to make three decisions for training and six decisions for the formal test. For each decision, two EPPs were displayed using MouselabWEB (Johnson et al., 2008) and participants were asked to freely access the information of the EPPs (see Figure 2A and a demonstrational video in Supplementary Materials), and then rate their preference on a nine point scale (see Appendix Figure A2), ranging from 1 (*definitely prefer Project A*) to 9 (*definitely prefer Project B*). They were told that the smaller the number they chose, the more they preferred Project A, the larger the number they chose, the more they preferred Project B, and choosing five meaning they have equal preference over the two projects. The experiment automatically ended when all the decisions were made.

The presentation of each choice (see Figure 2A) was the combination of two layers of contents, the Information Layer (see Figure 2B) and the Masking Layer (see Figure 2C). The Information Layer contained the details of the projects, where contents of the three attributes (i.e., MC, SR, and TC) were shown for each EPP.

Each of the three attributes had two levels, indicating how well or badly the EPP performs. In order to create the same scale for all the attributes, we used ranks to demonstrate superiority or inferiority. When an EPP performed well on one attribute, it was expressed as “better than 90% of all other projects” (*superior*), when it performed badly, it was expressed as “better than 10% of all other projects” (*inferior*).

The masking layer contained six boxes which concealed the contents of the attributes. The name of each attribute was written on the boxes. When the cursor is hovering over a box, it opens and reveals the content below (i.e., Figure 2A).

For each pair of EPPs, only one of the three attributes was set as the common feature, which can be either superior or inferior. For example, in Figure 2, TC was the common feature and set as superior (i.e., better than 90% of all other projects). The other two attributes were set as unique features and were balanced between the two EPPs (i.e., superior and inferior vs. inferior and superior). Since each of the three attributes could be a superior or inferior common feature in the whole experiment, six pairs of EPPs were generated in total (see Appendix Table A1 for detailed designs of the EPPs).

#### Manipulation and Measurement

##### General SI of common features

Based on the results from our preliminary study, we set TC (low [1] SI), MC (medium [2] SI), and SR (high [3] SI), respectively, as the common features, to test the effect of general SI on the level of consideration. Since both SR and EO had a high level of importance ratings and their difference was not significant, we used SR instead of EO because it had the highest importance rating.

##### Individual SI of common features

We used a method similar to Analytical Hierarchy Process (AHP; Saaty, 1980) to measure the individual SI of attributes in real decision-making. The logic of this approach is that, when facing a trade-off between two attributes, if an individual thinks one attribute is more important than the other, he/she should prefer the EPP with the more important attribute at superior level than the EPP with the less important attribute at superior level. For example, if one thinks SR as more important than MC, one will choose the project with superior SR and inferior MC rather than inferior SR and superior MC.

**FIGURE 3:**
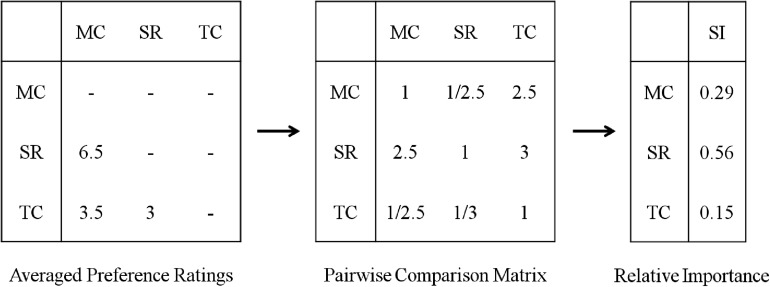
Demonstration of the calculation of individual subjective importance. MC, monetary cost; SR, success rate; TC, time cost; SI, subjective importance.

In this study, six pairs of EPPs were designed to create tradeoffs between each two of the three attributes (i.e., MC, SR, and TC). Participants faced a trade-off between each two attributes where the common feature was either inferior or superior. Therefore, by averaging the preference ratings between decisions with inferior or superior common features, three common-feature-insensitive ratings were acquired, which formed into a single loop of comparison among the three attributes. Then, the ratings were inserted into a 3 × 3 matrix and transformed into a pairwise comparison matrix, which represented the relative preferences between each two attributes (see Figure 3). The SI of the attributes was acquired by calculating the standardized eigenvector of the matrix. For each participant, the SI of the three attributes always summed to one and was assumed to remain constant for all six decisions. An R program for the calculation is also provided in Supplementary Materials. The individual SI of a common feature is the relative importance of the attribute serving as the common feature.

##### Level of consideration of common features

Two indicators (*Attention on common features* and *Impact of common features*) were used to measure the level of consideration of common features. The first indicator focused on measuring the attention that a common feature received from the decision maker. More attention indicates a higher level of consideration. The second indicator focused on measuring the absolute preference change (i.e., the impact on the decision) induced by changing the contents of common features. More preference change indicates higher level of consideration.

To measure participants’ attention on common features, we used MouselabWEB to display the EPPs and track participants’ mouse traces (see Figure 2). The content of MC, SR, and TC was covered with gray boxes and was only revealed when the cursor was hovering over the corresponding box. The open and close of a box, with hover time above 200 ms, was recorded as one valid information access count. The equation is

$$\mathrm{Attention}\mathrm{on}\mathrm{common}\mathrm{features}=\frac{{\text{IAC}}_{\text{CF}}}{{\text{IAC}}_{\text{AF}}}$$

where IAC_CF_ denotes the “valid information access counts on common features” (two boxes) and IAC_AF_ denotes the “valid information access counts on all features” (six boxes). The attention on common features ranges from 0 to 1. The larger the number is, the higher the level of consideration is.

To measure the impact of common features, we calculated the absolute preference change induced by changing the content of the common feature. The equation is

$$\mathrm{Impact}\mathrm{of}\mathrm{common}\mathrm{features}=\left|{\text{PR}}_{\text{CFS}}-{\text{PR}}_{\text{CFI}}\right|$$

where PR_CFS_ denotes the “preference rating with common feature at superior level” and PR_CFI_ denotes the “preference rating with common feature at inferior level.” The impact of common features could range from 0 to 8. The larger the number is, the higher the level of consideration is.

## Results

### Exclusion of Data

Six participants were excluded because their Mouselab data (i.e., valid information access counts) were partly or completely missing, which may be the result of irresponsible participation. The final sample size for hypothesis testing was 67.

### Descriptive Statistics

Table 1 shows descriptive statistics of major variables measured in Study 1. Consistent with the preliminary study, SR had the highest average individual SI among the three attributes, MC had the second highest, and TC had the third highest.

**TABLE 1:** Descriptive statistics of major variables in Study 1 (*N* = 67).

	General SI of common feature					
	Low (TC)		Medium (MC)		High (SR)	
	Mean	SD	Mean	SD	Mean	SD
Individual SI	0.19	0.08	0.27	0.08	0.54	0.09
Attention on common features	0.22	0.11	0.27	0.17	0.31	0.10
Impact of common features	1.03	1.31	1.04	1.26	1.76	1.81

### Hypothesis Testing

Because of the within-subject design, we used generalized estimating equations (GEE) to test the effect. The linear model was used, and the structure of working correlation matrix was set as exchangeable. The subject variable was participants’ id, and the within-subject variable was the general SI of common features.

Because people tend to read from top to bottom, when serving as the common feature, attributes at the top (e.g., MC) may naturally draw more attention than those at the bottom (e.g., TC). Therefore, a new factor, *position of common features*, was included as a covariate affecting attention on common features. This factor has three levels including top (3), middle (2), and bottom (1).

**FIGURE 4:**
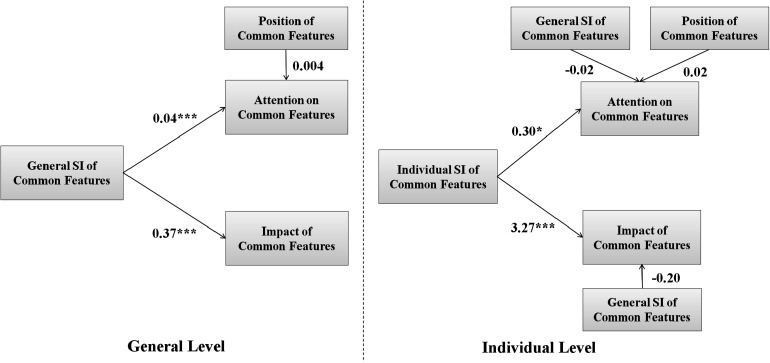
Path diagram of generalized estimating equations in Study 1. Unstandardized coefficients are shown. ^∗^*p* < 0.05, ^∗∗∗^*p* < 0.001.

As Figure 4 shows, on the general level, there is a significant and positive relation between the general SI of common features and the attention on common features (*B* = 0.04, β = 0.25, *Wald*χ^2^(1) = 15.15, *p* < 0.001, 95% CI = [0.02, 0.06]). There is also a significant and positive relation between the general SI and the impact of common features (*B* = 0.37, β = 0.20, *Wald*χ^2^(1) = 11.70, *p* < 0.001, 95% CI = [0.16, 0.58]). These results indicate that common features with higher SI received more attention and their change induced more preference change, thus having a higher level of consideration.

Moreover, on the individual level, there is a significant and positive relation between the individual SI of common features and the attention on common features (*B* = 0.30, β = 0.38, *Wald*χ^2^(1) = 6.46, *p* = 0.01, 95% CI = [0.07, 0.53]). There is also a significant and positive relation between the individual SI of common features and the impact of common features (*B* = 3.27, β = 0.37, *Wald*χ^2^(1) = 13.36, *p* < 0.001, 95% CI = [1.52, 5.02]). These results indicate that the level of consideration increased with the SI of the common features on the individual level, when controlling the general SI of the common feature.

### Discussion

In this study, we found that the level of consideration of common features did increase with their SI, and this effect held significant on both the general level and the individual level. This result provided good evidence to support our hypothesis. Yet, in the design of Study 1, we altered the SI of common features by setting attributes with different levels of importance as the common feature. This did not allow us to directly test if increasing the SI of the same common attribute would increase the level of consideration.

Therefore, to further test our hypothesis, we manipulated the SI of the common SR (i.e., when set SR as the common feature) by assigning different EO to the projects. Usually, when the EO of an EPP is at a higher level, SR would be more crucial for the decision maker to ensure the realization of the outcome. Therefore, we assumed that the SI of a common SR will be higher when the EO is at a higher level, which will further lead to a higher level of consideration of the common SR. This was tested in Study 2.

## Study 2

### Methods

#### Participants and Design

Seventy students (female: 27, male: 43, mean age = 23.71, SD = 0.92) from another local university (Zhejiang University) volunteered to participate in this experiment. All participants were recruited via online notices in different QQ (i.e., widely used online communication software) groups and the BBS of the university. The participation was voluntary and there were no monetary or any form of compensations.

All participants finished the experiment in our computer lab. A 3 × 2 mixed design was used in this study (within-subject variable: General SI of Common Features [Low SI feature: TC; Medium SI feature: MC; High SI feature: SR]; Between-subject variable: EO, Small [n = 39] vs. Large [n = 31]; Dependent variable: level of consideration of common features).

#### Procedure and Material

Each participant was randomly assigned to one level of EO. In the experiment, participants first finished three decisions for training and answered four questions to check their understanding of the decision task (*manipulation check*). Participants then finished six formal decisions. In the end, they were asked to recall the EO of the EPPs in their experiment (*manipulation check*). The rest of the details were identical to those in Study 1.

#### Manipulation and Measurement

All the details were identical to those in Study 1 except two additional manipulations.

##### Level of Environmental Outcome

**FIGURE 5:**
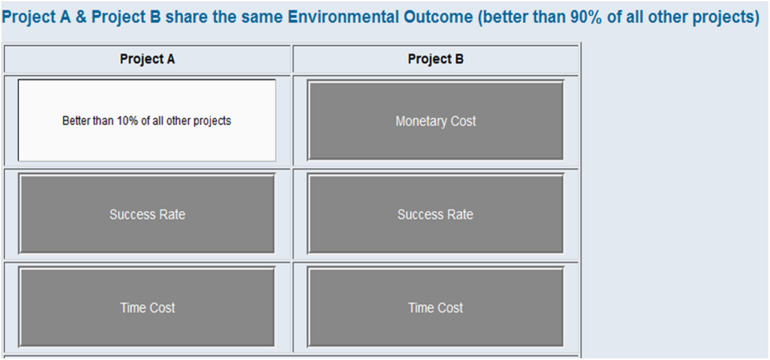
Demonstration of the decision task in Study 2.

We manipulated the level of EO by setting it as “better than 10% of all other projects” (Small) or “better than 90% of all other projects” (Large) for all the EPPs. The information was presented above each pair of the EPPs in every decision (see Figure 5).

##### Manipulation Check

We included two manipulation checks to exclude data from careless participation. In the first check, participants were asked to answer four questions to check their understanding of the decision task (see Supplementary Materials). Participants were warned and required to answer again, when they submitted incorrect answers. Those who answered incorrectly for too many times (above seven times) were excluded in the analysis. In the second check, participants were asked to recall the EO of the EPPs in their experiment and choose from three possible alternatives, including better than 10, 50, or 90% of all other projects. Those who answered incorrectly were excluded in the analysis.

### Results

#### Exclusion of Data

One participant was excluded for failing the first manipulation check. Eight additional participants were excluded for failing the second manipulation check. Three additional participants were also excluded because their Mouselab data were partly or completely missing. The final sample size for hypothesis testing was 58 [EO: Small (*n* = 32) vs. Large (*n* = 26)].

#### Descriptive Statistics

Table 2 shows descriptive statistics of major variables measured in Study 2. The results were consistent with Study 1.

**TABLE 2:** Descriptive statistics of major variables in Study 2 (*N* = 58).

		Common feature					
		Monetary cost		Success rate		Time cost	
		Mean	SD	Mean	SD	Mean	SD
Small environmental outcome	Subjective importance	0.28	0.08	0.53	0.10	0.19	0.08
	Attention on common features	0.29	0.09	0.33	0.11	0.24	0.07
	Impact of common features	1.00	1.39	1.41	1.19	0.97	1.03
Large environmental outcome	Subjective importance	0.23	0.11	0.61	0.09	0.16	0.05
	Attention on common features	0.33	0.10	0.39	0.09	0.21	0.07
	Impact of common features	0.69	0.74	2.42	2.45	1.00	1.60

#### Hypothesis Testing

The first two hypotheses tested in Study 2 were the same as those in Study 1. The third hypothesis was added by manipulating the SI of the common SR with different levels of EO, and to test its effect on common feature consideration directly. We hypothesized that the SI of a common SR will be higher when the EO is at a higher level, which will further lead to a higher level of consideration of the common SR.

**FIGURE 6:**
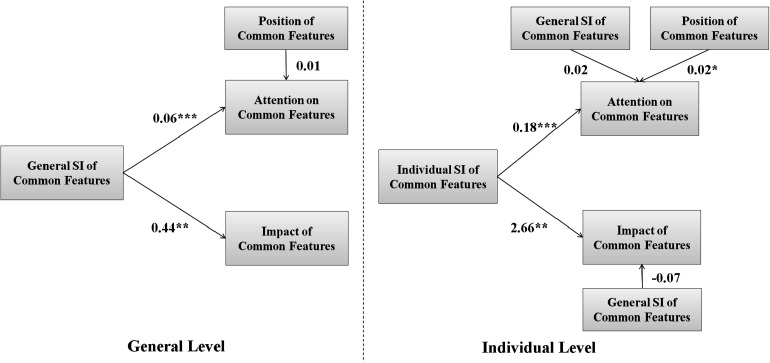
Path diagram of generalized estimating equations in Study 2. Unstandardized coefficients are shown. ^∗^*p* < 0.05, ^∗∗^*p* < 0.01, ^∗∗∗^*p* < 0.001.

As Figure 6 shows, on the general level, there is a significant and positive relation between the general SI of common features and the attention on common features (*B* = 0.06, β = 0.48, *Wald*χ^2^(1) = 31.39, *p* < 0.001, 95% CI = [0.04, 0.08]). There is also a significant and positive relation between the general SI and the impact of common features (*B* = 0.44, β = 0.23, *Wald*χ^2^(1) = 8.02, *p* = 0.005, 95% CI = [0.14, 0.74]). These results indicate that our hypothesis is supported on the general level.

On the individual level, there is a significant and positive relation between the individual SI of common features and the attention on common features (*B* = 0.18, β = 0.33, *Wald*χ^2^(1) = 11.87, *p* < 0.001, 95% CI = [0.08, 0.28]). There is also a significant and positive relation between the individual SI of common features and the impact of common features (*B* = 2.66, β = 0.34, *Wald*χ^2^(1) = 10.05, *p* = 0.002, 95% CI = [1.02, 4.31]). These results indicate that our hypothesis is supported on the individual level.

**FIGURE 7:**
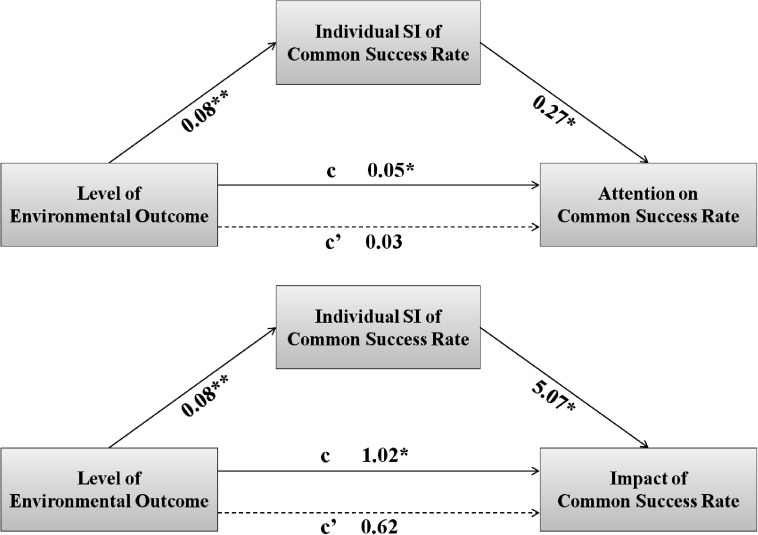
Path diagram of mediation model in Study 2. Unstandardized coefficients are shown. *p < 0.05, **p < 0.01.

Moreover, as Figure 7 shows, we tested the mediation effect of individual SI of common SR. We used PROCESS for SPSS (Preacher and Hayes, 2008) for the test. Because our goal was to test whether the manipulation of EO (i.e., independent variable) would change the SI of common SR (i.e., mediator), which in turn changed its level of consideration (i.e., dependent variable). Only two models (Model 4 and Model 74) were suitable. We chose the simple mediation model (Model 4) because it required fewer assumptions and there were no reasons to assume a moderation between the independent variable and the mediator. Nevertheless, we provided the results of Model 74 in the Supplementary Materials. We set the bootstrap samples to 5,000 and the bias-corrected confidence level to 95%.

The results showed that the mediation effect was significant and positive for both the attention on common SR (indirect effect = 0.02, 95% CI = [0.00, 0.05]) and the impact of common SR (indirect effect = 0.39, 95% CI = [0.10, 0.83]). This result further supported our hypothesis and indicated that increasing the SI of the same common attribute did increase the level of consideration.

## General Discussion

This article provides a strong evidence that the level of consideration will increase with the SI of common features, and this result holds significant for different indicators on both the general level and the individual level. Together, it indicates that there is a top-down approach for explaining how people consider the common feature in multi-attribute decision-making and that SI of common features should be one of essential determinants.

### Novelty and Implications

Our research is novel in two ways. First, we provided evidence for a new explanation on what affects the consideration of common features. Our findings indicate that SI of common features can decide their consideration. This is because individuals pay more attention on common features that they view as more important, which is consistent with studies about visual attention (Theeuwes, 2010; Orquin and Loose, 2013).

Moreover, it is possible that the high priority and attention will encourage decision makers to center on the important common feature when evaluating the alternatives (Anderson et al., 2011). This, in turn, may cause different evaluation of the unique features (e.g., giving more value to high SR when the stake [common MC] is higher and important), which gives the common feature more impact on the decision. Together, as a theoretical implication, our findings connected these two effects and provided insights on the underlying process of multi-attribute decision-making.

Secondly, our findings explained a wider range of the effect of common feature cancelation. Opposite to previous studies following the bottom-up approach, finding independent characteristics (e.g., salience) of common features (Du and MaCdonald, 2015), we followed the top-down approach and focused on including the decision maker in our explanation. As another implication, this finding helped us to investigate the effect on the individual level and provide insights in explaining effects like individual difference (e.g., some people may ignore the near expiration date because it is not important to them, and choose the 1-gallon bottle of milk over the 1-liter bottle, causing a substantial waste).

### Limitations and Future Research Orientation

Our research also has its limitations, and hopefully, these limitations may facilitate new lines of research in the future. One limitation is that we tested the effect of SI of common features with multiple indicators on multiple levels. Yet, like the effect of interdependence, the effect of SI may also have constraints and moderators (Li et al., 2007; Su et al., 2012). For example, the effect may be larger when a decision is harder to make, based on the content of unique features (i.e., when neither of the two options dominate the other based on the content of unique features). Because for a hard decision, people will seek extra information for help and pay more attention to the important common features. However, for an easy decision, people may generally ignore all common features regardless of their importance. Therefore, future research may focus on the constraints and moderators of the effect of SI.

Another limitation is that we did not further investigate the connection between the top-down approach and the bottom-up approach in our research. Previous studies suggested that individuals’ attention should be affected through both the bottom-up approach (salience and interdependence of common features) and the top-down approach (individuals’ SI). Therefore, it would be interesting to check whether there are any connections between a top-down factor and a bottom-up factor. For instance, a common feature may be viewed as subjectively more important when presented in salient form (large font size), rather than an ordinary form (small font size), because people may read between the lines and suspect that the information provider would want to highlight their own important features. Hence, future research can focus on investigating the possible interactions between top-down and bottom-up factors.

## Data Availability Statement

The original contributions presented in the study are included in the article/Supplementary Material, further inquiries can be directed to the corresponding author/s.

## Ethics Statement

The studies involving human participants were reviewed and approved by the Ethics Committee of Psychology and Behavioral Sciences, Zhejiang University. Written informed consent for participation was not required for this study in accordance with the national legislation and the institutional requirements.

## Author Contributions

ZW and GH designed the experiments. ZW conducted the experiments and performed the analysis of the data. ZW and GH wrote and revised the manuscript. Both authors contributed to the article and approved the submitted version.

## Conflict of Interest

The authors declare that the research was conducted in the absence of any commercial or financial relationships that could be construed as a potential conflict of interest.

## Appendix

**TABLE A1:** Design of six pairs of EPPs in the two studies.

		Project A				Project B			
Group	Question number	Monetary cost	Success rate	Time cost	Environmental outcome	Monetary cost	Success rate	Time cost	Environmental outcome
A	1	90%	10%	***10%***	***10%***	10%	90%	***10%***	***10%***
	2	90%	10%	***90%***	***10%***	10%	90%	***90%***	***10%***
	3	90%	***10%***	10%	***10%***	10%	***10%***	90%	***10%***
	4	90%	***90%***	10%	***10%***	10%	***90%***	90%	***10%***
	5	***10%***	90%	10%	***10%***	***10%***	10%	90%	***10%***
	6	***90%***	90%	10%	***10%***	***90%***	10%	90%	***10%***
B	1	90%	10%	***10%***	***90%***	10%	90%	***10%***	***90%***
	2	90%	10%	***90%***	***90%***	10%	90%	***90%***	***90%***
	3	90%	***10%***	10%	***90%***	10%	***10%***	90%	***90%***
	4	90%	***90%***	10%	***90%***	10%	***90%***	90%	***90%***
	5	***10%***	90%	10%	***90%***	***10%***	10%	90%	***90%***
	6	***90%***	90%	10%	***90%***	***90%***	10%	90%	***90%***

*(1) 10%: better than 10% of all other projects; 90%: better than 90% of all other projects, (2) Common features were **blackened and italic** in this table. (3) Study 1 only has one group and does not have the content of environmental outcome. Unstandardized coefficients are shown. *p < 0.05, **p < 0.01, ***p < 0.001.*
